# Supplementary material for: Enhanced Mechanical Properties of Multiscale Carbon Fiber/Epoxy Unidirectional Composites with Different Dimensional Carbon Nanofillers
Source: Nanomaterials (Basel). 2020 Aug 26;10(9):1670. doi: 10.3390/nano10091670 (PMC7558300; doi:10.3390/nano10091670)
Supplement: Supplementary file 1 [file nanomaterials-10-01670-s001.pdf]

nanomaterials

Article

# Enhanced Mechanical Properties of Multiscale Carbon Fiber/Epoxy Unidirectional Composites with Different Dimensional Carbon Nanofillers

**Yu Liu [1], Dong-Dong Zhang [2], Guang-Yuan Cui [1], Rui-Ying Luo [1,*] and Dong-Lin Zhao [2,*]**

1 Research Institute for Frontier Science, Beihang University, Beijing 100029, China; liuyu9175@buaa.edu.cn (Y.L.); m15300178689_3@163.com (G.-Y.C.)
2 State Key Laboratory of Chemical Resource Engineering, Key Laboratory of Carbon Fiber and Functional Polymers, Ministry of Education, Beijing University of Chemical Technology, Beijing 100029, China; zhangdd8888@126.com
* Correspondence: ryluo@buaa.edu.cn (R.-Y.L.); dlzhao@mail.buct.edu.cn (D.-L.Z.)

Relevant spots for the 3% sample are as follows.

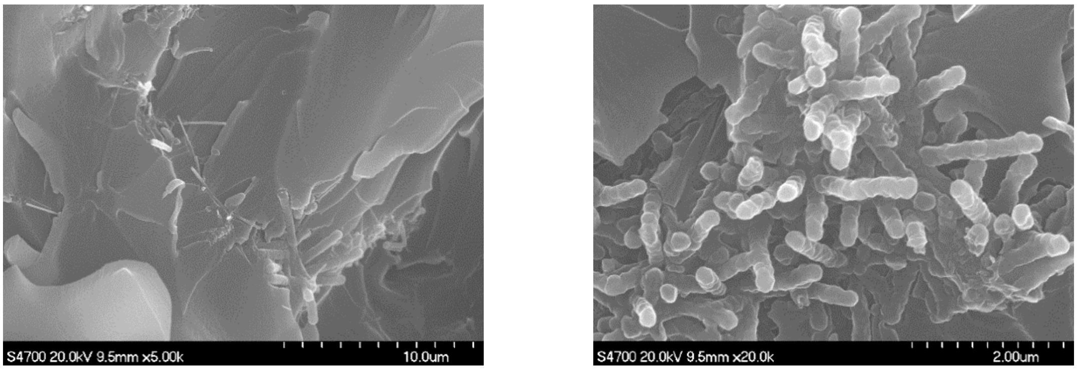


(**a**) (**b**)

**Figure S1.** SEM micrographs of the epoxy composites with AMGNS-MWCNT mixture contents of 3.0 wt%. (**a**) the fractured surface of the composites; (**b**) the agglomeration CNTs in matrix of the composites.

More SEM images of the fractured surface of the epoxy composites with AMGNS-MWCNT mixture contents of 1.0 wt% and 2.0 wt% are as follows.

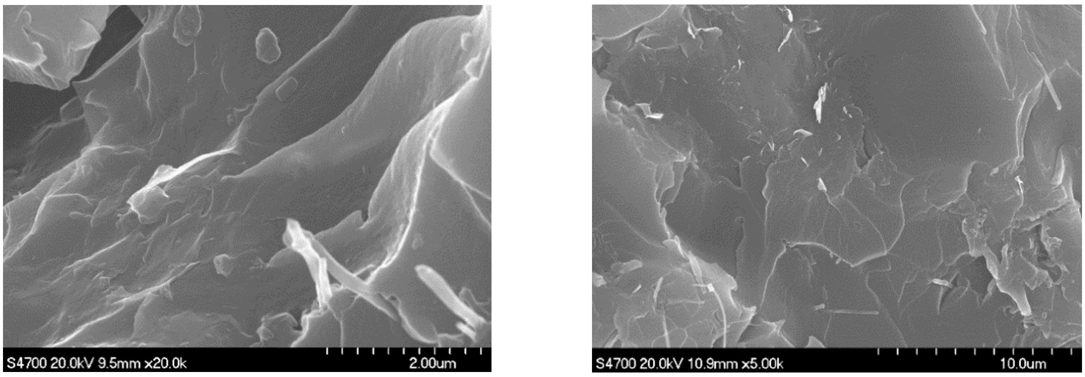


(**a**) (**b**)

**Figure S2.** SEM micrographs of the fractured surface of the epoxy composites with AMGNS-MWCNT mixture contents of (**a**) 1.0 wt%, (**b**) 2.0 wt%.

GNS can be seen in AMGNS-MWCNT/epoxy composites in Figure 4(a) and (b).

© 2020 by the authors. Licensee MDPI, Basel, Switzerland. This article is an open access article distributed under the terms and conditions of the Creative Commons Attribution (CC BY) license (http://creativecommons.org/licenses/by/4.0/).
